# Non-SMC elements 1 and 3 are required for early embryo and seedling development in Arabidopsis

**DOI:** 10.1093/jxb/erx016

**Published:** 2017-02-16

**Authors:** Gang Li, Wenxuan Zou, Liufang Jian, Jie Qian, Yingtian Deng, Jie Zhao

**Affiliations:** 1 State Key Laboratory of Hybrid Rice, College of Life Sciences, Wuhan University, Wuhan 430072, China

**Keywords:** Arabidopsis, development, early embryo, mitosis, non-SMC element, organ, seedling

## Abstract

Early embryo development from the zygote is an essential stage in the formation of the seed, while seedling development is the beginning of the formation of an individual plant. AtNSE1 and AtNSE3 are subunits of the structural maintenance of chromosomes (SMC) 5/6 complex and have been identified as non-SMC elements, but their functions in Arabidopsis growth and development remain as yet unknown. In this study, we found that loss of function of *AtNSE1* and *AtNSE3* led to severe defects in early embryo development. Partially complemented mutants showed that the development of mutant seedlings was inhibited, that chromosome fragments occurred during anaphase, and that the cell cycle was delayed at G2/M, which led to the occurrence of endoreduplication. Further, a large number of DNA double-strand breaks (DSBs) occurred in the *nse1* and *nse3* mutants, and the expression of *AtNSE1* and *AtNSE3* was up-regulated following treatment of the plants with DSB inducer compounds, suggesting that *AtNSE1* and *AtNSE3* have a role in DNA damage repair. Therefore, we conclude that *AtNSE1* and *AtNSE3* facilitate DSB repair and contribute to maintaining genome stability and cell division in mitotic cells. Thus, we think that *AtNSE1* and *AtNSE3* may be crucial factors for maintaining proper early embryonic and post-embryonic development.

## Highlight

AtNSE1 and AtNSE3 are crucial factors for early embryo and seedling development, and mutations of *AtNSE1* and *AtNSE3* can affect cell division and the DNA repair process.

## Introduction

Double fertilization is a crucial developmental process in angiosperms. The embryo and endosperm, as the products of double fertilization, are the major constituents of early seeds in Arabidopsis (Bleckmann *et al.*, 2014). Beginning with a single-celled zygote, the embryo undergoes a highly ordered sequence of cell divisions during which the new emerging tissues are specified and patterned. At the same time, the endosperm, which is produced by the fusion of a central cell and a sperm cell, undergoes a series of mitoses, developing into the syncytial endosperm. Subsequently, the endosperm is cellularized and degraded gradually in the later stages of embryogenesis (Li and Berger, 2012; Sreenivasulu and Wobus, 2013). During this, the process of early embryo development is a key step (Xu *et al.*, 2004; Jeong *et al.*, 2011).

Genome stability is of crucial importance for living organisms. It is known that DNA damage occurs throughout the life cycle of plants. In contrast to the lesions that disturb only one DNA strand, double-strand breaks (DSBs) pose a particularly damaging threat to genome stability. Even a single DSB can cause cell death. In somatic cells, DSBs can arise not only due to internal events such as replication and transposon excision, but also due to interactions with radiation or genotoxic compounds (Knoll *et al.*, 2014). Thus, DSB repair is essential for the survival of all organisms. In current theory, DSBs can be repaired via two main pathways: non-homologous end joining (NHEJ) and homologous recombination (HR) (Puchta, 2005). NHEJ is the main mode of DSB repair in somatic cells, and is typically thought to involve three different mechanisms, namely classical or canonical NHEJ (cNHEJ), alternative NHEJ (aNHEJ), and microhomology-mediated end joining (MMEJ). In Arabidopsis, *AtKu80*, *AtXrcc1*, and *AtXpf* have been shown to be involved, respectively, in these three mechanisms (Mladenov and Iliakis, 2011; Charbonnel *et al.*, 2011; Jia *et al.*, 2013). Additionally, three repair subpathways of HR have been discovered in plant cells: classical double-strand break repair (DSBR), synthesis-dependent strand annealing (SDSA), and single-strand annealing (SSA). In yeast, a separate ‘break-induced replication’ (BIR) pathway has been described (Bray and West, 2005; Llorente *et al.*, 2008; Pardo *et al.*, 2009). DSBR, which leads to gene conservation and crossover events, is only involved in meiosis, while SDSA and SSA are the major mechanisms that repair DSBs in somatic cells. It has been demonstrated that *AtRAD51*, *AtRAD51C*, *AtXRCC3*, and *AtRAD54* are involved in homologous recombination in the SDSA pathway (Roth *et al.*, 2012). It is also known that some DNA helicases and nucleases, such as AtRECQ4A, AtFANCM, and AtMUS81, also play important roles in SDSA (Mannuss *et al.*, 2010; Roth *et al.*, 2012). Recently, it has been reported that the AtRAD51 paralogues AtXRCC2, AtRAD51B, and AtRAD51D can participate in the SSA pathway (Da Ines *et al.*, 2013). Although many regulators have been characterized in the DSB repair pathways, the exact events that occur in these repair mechanisms still require fuller elucidation.

Structural maintenance of chromosome (SMC) proteins are conserved chromosomal ATPases that regulate nearly all aspects of chromosome biology during both meiosis and mitosis; they are crucial for genome stability. There are six SMC proteins (SMC1–6) in eukaryotes, forming three distinct SMC complexes: cohesin (including the core of SMC1/3), condensin (including the core of SMC2/4), and the SMC5/6 complex. It is known that cohesin and condensin play critically important roles in, respectively, sister-chromatid cohesion and chromosome condensation. These SMC complexes are also involved in DNA repair and gene regulation (Nasmyth and Haering, 2009; Hirano, 2012). The SMC5/6 complex, which does not yet have a functionally descriptive name, has been implicated mainly in DNA repair, but has also been associated with chromosome replication and segregation (Murray and Carr, 2008; De Piccoli *et al.*, 2009; Kegel and Sjögren, 2010; Wu and Yu, 2012). It contains the SMC5 and SMC6 proteins, as well as non-SMC elements (NSEs) including NSE1, NSE2/MMS21, NSE3/MAGE-G1, NSE4, NSE5, and NSE6 (Palecek *et al.*, 2006; Pebernard *et al.*, 2006; Duan *et al.*, 2009; Yan *et al.*, 2013; Räschle *et al.*, 2015). NSE1 contains a RING finger domain that is typical of ubiquitin ligases, and is required for SMC5/6 holocomplex integrity in yeast (Fujioka *et al.*, 2002; McDonald *et al.*, 2003; Pebernard *et al.*, 2004; Santa Maria *et al.*, 2007). NSE2/MMS21 has SUMO ligase activity, required for DNA damage repair (Pebernard *et al.*, 2004; Potts and Yu, 2005; Zhao and Blobel, 2005; Andrews *et al.*, 2005). NSE3 can form a subcomplex with NSE1 and NSE4, and plays an important role in meiosis in yeast (Pebernard *et al.*, 2004, 2008; Hudson *et al.*, 2011; Kozakova *et al.*, 2015). In Arabidopsis, mutation of *SMC5* and *SMC6A/6B* caused defects in sister chromatid alignment and homologous recombination after DNA damage (Watanabe *et al.*, 2009). Another study showed that the SMC5/6 complex is required for the repair of DNA damage induced by the cytidine analog zebularine (Liu *et al.*, 2015). It is known that AtMMS21 interacts with AtSMC5 and that they act in repairing DSBs, in stem cell niche maintenance during root development, and in gametophyte development (Huang *et al.*, 2009; Zhang *et al.*, 2010; Xu *et al.*, 2013; Yuan *et al.*, 2014; Liu *et al.*, 2014*a*). *ASAP1* and *SNI1* were identified as *NSE5* and *NSE6* in Arabidopsis (Yan *et al.*, 2013). SNI1 is a negative regulator of NPR1, which is involved in plant immune responses (Yan *et al.*, 2013). In addition, *AtNSE1* was characterized as an embryo defective gene, and also identified as *EMB1379* (Tzafrir *et al.*, 2004). However, the functions of *AtNSE1* are as yet unclear in Arabidopsis.

Here, we demonstrate that *AtNSE1* and *AtNSE3* are essential for early embryogenesis and post-embryonic development. We provide evidence that mutations of *AtNSE1* or *AtNSE3* caused disorderly cell division in early embryos and seedlings, leading to seed abortion and seedling lethality. We found that mitosis displayed some defects in the mutant somatic cells, including chromosome missegregation, cell cycle delay in G2/M and occurrence of endoreduplication. In addition, the mutants of *AtNSE1* and *AtNSE3* could affect DSB repair and displayed more sensitivity to DSB damage than wild-type. Our results establish that both *AtNSE1* and *AtNSE3*, through their functions in participating in DNA damage repair, play crucial roles in early embryo, endosperm, and post-embryonic seedling development.

## Materials and methods

### Plant materials and growth conditions

In this study, *Arabidopsis thaliana* ecotype Columbia (Col) was used as the test material. The T-DNA insertion mutants CS16151 (*nse1-1/+*), CS24066 (*nse1-2/+*), and CS334183 (*nse3-2/+*, an individual line obtained from a set of lines CS451171) were obtained from the Arabidopsis Biological Resource Center (ABRC, Ohio State University, http://abrc.osu.edu/), and N734712 (*nse3-1/+*) was obtained from Nottingham Arabidopsis Stock Centre (NASC, University of Nottingham, http://arabidopsis.info/).

All plants were grown in a greenhouse at Wuhan University at 22 ± 2 °C with a 16 h light–8 h dark photoperiod.

### Complementation analysis

For complementing the mutants, the promoters and coding sequences of *AtNSE1* and *AtNSE3* were amplified with KOD-Plus-Neo DNA polymerase (Toyobo) from wild-type Arabidopsis and cloned into *mpCambia1300-eGFP* vector, and then introduced into *nse1-1/+*, *nse1-2/+*, and *nse3-1/+*, *nse3-2/+* heterozygote mutants, respectively, via a floral dip method (Clough and Bent, 1998). The primers used in the experiments are listed in Supplementary Table S1 at *JXB* online.

For partial complementation, a 5197bp *ABI3* promoter was used to construct *ABI3::NSE1-GFP* and *ABI3::NSE3-GFP*. The *ABI3* promoter was obtained from the wild-type genome by PCR, and was inserted into *pCAMBIA1300* with *Pst*I and *Kpn*I, resulting in *ABI3*-*pCAMBIA1300*. The fragments NSE1-GFP-NosT and NSE3-GFP-NosT were obtained from *pNSE1::NSE1-GFP* and *pNSE3::NSE3-GFP*, respectively, by double digestion with *Kpn*I and *Eco*RI, and were inserted into *ABI3-pCAMBIA1300*. All the constructs were transferred into *Agrobacterium tumefaciens* strain GV3101, which was used to transfer into the *nse1-1/+* or *nse3-1/+* mutant by the floral-dip method. The obtained transgenic progenies were screened on hygromycin plates and identified by PCR. The homologous transgenic lines *ABI3::NSE1 nse1-1/+* and *ABI3::NSE3 nse3-1/+* were used for subsequent analysis.

### Root growth assays

To measure root length, roots were laid on a plate and imaged with a Nikon D5000. To measure the length of root apical meristem (RAM), roots were mounted onto microscope slides with Hoyer’s solution for 2–4 h, and the cleared roots were examined by differential interference contrast microscopy under an inverted microscope (Olympus TH4-200) equipped with a CCD of a SPOT digital microscope camera (Diagnostic Instruments). Quantification of root length was performed using Digimizer software (http://www.digimizer.com/). The experiment was repeated at least three times.

### Ovule clearing and observation of endosperm cellularization

Fresh ovules were dissected from siliques using forceps and mounted in Hoyer’s solution (chloral hydrate:glycerol:water, 8:1:2 (w/v/v)) for 30 min to 6–8 h depending on the embryo developmental stage (Chen *et al.*, 2015). Then, the cleared ovules were observed and photographed with differential interference contrast microscopy (Olympus TH4-200 equipped with a CCD of a SPOT digital microscope camera).

To observing endosperm cellularization, we used a reported method (Liu *et al.*, 2014*b*) modified as followed. The fresh siliques were harvested and fixed in 4% glutaraldehyde in PBS (pH 7.0). After being vacuumed until all pods were sunk in the fixative, the material was placed into fresh fixative and fixed overnight at room temperature. Next, the samples were dehydrated and rehydrated by a series of graded alcohols for 20 min for each gradient. Finally the ovules were dissected from the rehydrated siliques using forceps, and mounted onto the slides with Hoyer’s solution until the tissue was cleared, then observed with 488 nm excitation under a confocal laser scanning microscope (Olympus FluoView FV1000).

### Quantitative RT-PCR

For gene expression pattern, quantitative RT-PCR (qRT-PCR) was carried out using SYBR Green fluorescence with a Rotor-Gene Q real-time PCR machine (Qiagen) (Zhong and Simons, 1999). The relative expression levels were analysed as described previously (Ma and Zhao, 2010). For differential expression of the genes, real-time PCR was performed using TransStart Top Green qPCR SuperMix (TransGen, China) with a Bio-Rad CFX Manager 3.1. The threshold cycle (*C*_t_) value was automatically calculated by the Bio-Rad CFX Manager 3.1 system software and the ΔΔ*C*_t_ method was used to calculate the relative expression levels (Pfaffl, 2001). An internal gene, GAPDH, was used to normalize the expression of genes in various RNA samples. Three independent biological replicates and three technical replicates of each sample were made for quantitative PCR analysis. Primers used in the experiments are listed in Supplementary Table S1.

### RNA *in situ* hybridization

Ovules of wild-type plants at various developmental stages were collected, and fixed and embedded in Paraplast Plus embedding medium as described previously. The antisense and sense probes used in the experiments were all generated by PCR amplification with T7 promoter adding primers (primers are listed in Supplementary Table S1), and followed by *in vitro* transcription with the DIG RNA Labeling Kit (SP6/T7; Roche, http://www.roche.com) according to the manufacturer’s instructions. The procedures of fixing, embedding, sectioning and the other procedures of RNA *in situ* hybridization were performed as described previously (Brewer *et al.*, 2006; Deng *et al.*, 2014). The samples were mounted with a coverslip and subsequently observed under an Olympus BX60 microscope, then photographed with the Olympus DP72 CCD.

### Yeast two-hybrid and bimolecular fluorescence complementation assays

The full-length open reading frames (ORFs; without stop codons) of *AtNSE1* and *AtNSE3* were subcloned into the *pGADT7* and *pGBKT7* vector separately. Yeast two-hybrid assay was performed according to the reported method in our lab (Deng *et al.*, 2014). For bimolecular fluorescence complementation (BiFC) assay, the full length ORFs (without stop codons) of *AtNSE1* and *AtNSE3* were inserted into the vectors *pCAMBIA-SPYNE* and *pCAMBIA-SPYCE* separately, and the assay performed according to Sparkes’ method (2006). Primers used in this test are listed in Supplementary Table S1.

### Flow cytometry analysis

For flow cytometry analysis, at least 10 000 nuclei isolated from the first pair of leaves of 10-day-old seedlings were used for each experiment. Nuclei isolation was performed according to a reported method (Li *et al.*, 2005). RNase (10 μg mL^–1^) was added to the filtered supernatant, and incubated on ice for 10 min. Then 50 μg mL^–1^ propidium iodide (PI) was added into the mixture above, and stained for 20 min. The relative fluorescence intensities were recorded with a Beckman flow cytometer and analysed by the software Summit 4.3. For the ploidy measurement, the endoreduplication index (EI) was calculated as EI=(0×%2C)+(1×%4C)+(2×%8C)+(3×%16C) (Sterken *et al.*, 2012) and averaged over at least three technical replicates.

### Cell viability assays

To detect dead cells in root tip, the seeds were germinated and cultured on non-selective 1/2 MS medium for 5 d; the roots of seedlings were mounted onto glass slides with 40 μg mL^–1^ PI, and then observed and imaged under a confocal microscope (Olympus FluoView FV1000).

### Determination of mitosis index

Roots were fixed in a solution of 4% paraformaldehyde in PBS for 40–50 min at room temperature, then washed three times for 5 min in PBS, and digested for 30 min in a 0.3% (w/v) cellulose R-10, 0.3% (w/v) macerozyme R-10 prepared in PBS. Then, the roots were washed three times for 5 min in PBS and mounted under coverslips. The samples were crushed, snap frozen with liquid nitrogen to remove the coverslip, and mounted in Vectashield (Vector Laboratories, Burlingame, CA, USA) containing 1 μg mL^–1^ 4′,6-diamidino-2-phenylindole (DAPI). They were analysed for mitotic stages under a confocal microscope (Olympus FluoView FV1000).

### Commet assay analysis

The true leaves of seedlings at 10 days after germination (DAG), cultured on 1/2 MS plates without DNA damage inducer, were chopped with a razor in a Petri dish kept on ice and containing 500 μL of 1×PBS plus 20 mM EDTA. The resulting mixture was filtered through a 60 μm nylon mesh twice. Fifty microliters of nuclei was mixed with 50 μl of 1% low melting point agarose (warmed at 37 ℃) and placed onto a microscopic slide with 1% normal melting point agarose. Nuclei were then unwound and subjected to electrophoresis according to the N/N protocol described by Menke *et al.* (2001). Then the slides were stained with 40 μg mL^–1^ PI and examined by epifluorescence microscopy (BX60, Olympus). DNA damage was calculated by averaging the values of the percentage of DNA in tails from three individual slides, scoring 80 comets per slide. The comet analysis was performed using Comet Score software (http://www.autocomet.com). The statistical significance was evaluated by Student’s *t*-test.

### RNA-seq analysis

Ovules at 7 days after pollination (DAP) were collected from wild-type, *nse1-1* and *nse3-1*. Preparation and sequencing of RNA were completed by Oebiotech Company. Total RNA was extracted using Trizol reagent (Invitrogen, USA) following the manufacturer’s protocol. RNA integrity was confirmed using a 2100 Bioanalyzer (Agilent Technologies). The samples for transcriptome analysis were prepared using Illumina’s kit following the manufacturer’s recommendations. The cDNA library was sequenced on the Illumina sequencing platform (HiSeqTM 2500) and 125 bp paired-end reads were generated. Raw data (raw reads) of fastq format were first processed using the NGS QC Toolkit (Patel and Jain, 2012). Sequencing reads were mapped to the Arabidopsis TAIR 10.0 reference genome using Tophat (http://tophat.cbcb.umd.edu/) with default parameters slightly modified. The FPKM and count value were calculated using eXpress (Mortazavi *et al.*, 2008). Differential expression analysis was performed using the DESeq R package. *P*<0.05 was set as the threshold for significance. Venny and gene ontology (GO) analysis were performed by Venny 2.1 (http://bioinfogp.cnb.csic.es/tools/venny/) and WEGO (Web Gene Ontology Annotation Plot; http://wego.genomics.org.cn/cgi-bin/wego/index.pl), respectively.

## Results

### Knock-out of *AtNSE1* and *AtNSE3* caused defects in seed development

**Fig. 1.**
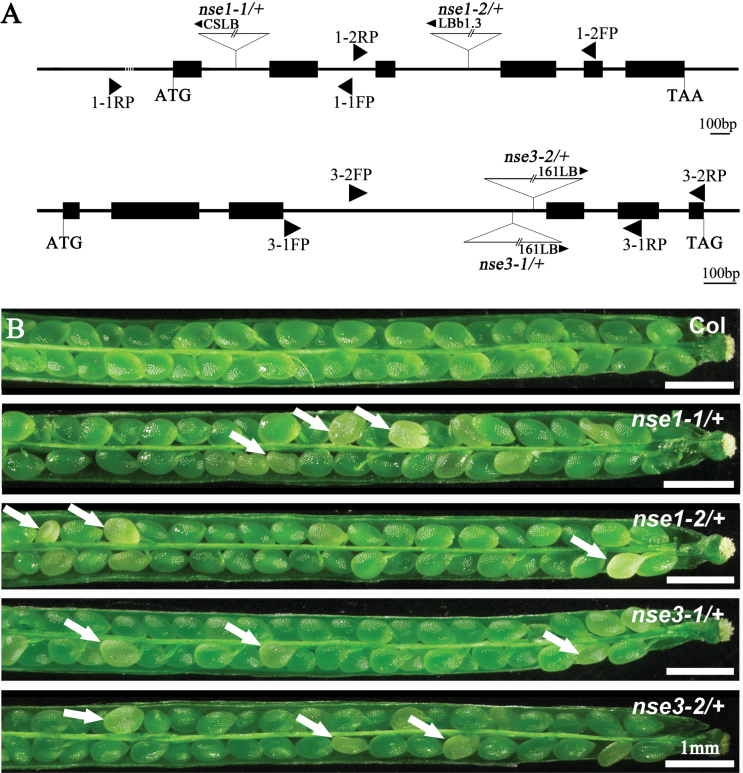
Characterization of the *AtNSE1* and *AtNSE3* mutations. (A) Schematic diagrams of the *AtNSE1* and *AtNSE3* gene structures with the positions of the T-DNA insertions of four mutants. Exons are shown as black boxes, and 5′ regions, 3′ regions and introns as lines. FP, forward primer; LB, left border primer; RP, reverse primer. (B) The silique phenotype of wild-type (Col), *nse1-1/+*, *nse1-2/+*, *nse3-1/+* and *nse3-2/+*. The white arrows show the aborted white ovules. Bars: 1 mm.

To investigate the function of *AtNSE1* and *AtNSE3* in Arabidopsis, we obtained four Arabidopsis T-DNA insertion mutant lines from public mutant collections: *nse1-1* (CS16151), *nse1-2* (CS24066), *nse3-1* (N734712), and *nse3-2* (CS334183) (Fig. 1A). We found that there were no viable homozygous mutant plants in the progenies of *nse1-1/+* and *nse1-2/+* plants (Supplementary Fig. S1A), and a similar result was obtained in *nse3-1/+* and *nse3-2/+* plants (Supplementary Fig. S1A), suggesting that the plants of homozygote genotype may be lethal. To confirm this, we dissected maturing siliques from *nse1-1/+*, *nse1-2/+*, *nse3-1/+*, and *nse3-2/+* plants to analyse the phenotypes, and found that all of them contained a proportion of aborted white seeds; this phenotype was not observed in wild-type plants (Fig. 1B). We further calculated the ratio of aborted seeds in each mutant and found that the phenotypic ratios for the aborted seeds were close to the expected values of 25% (Table 1). These results suggest that mutations of *AtNSE1* or *AtNSE3* can lead to lethality of homozygous progenies. Segregation analyses for all four of the self-fertilized mutant progenies were conducted. As it lacked kanamycin resistance, we analysed *nse1-2* plants for T-DNA insertions by PCR; the other mutants were analysed with resistance screening (BASTA for *nse1-1*, sulfadiazine for *nse3-1* and *nse3-2*). All of the segregation ratios (T-DNA:without T-DNA) were about 2:1, rather than the expected 3:1 (Table 2). This result indicated that all of the mutants contained a single copy T-DNA insertion in their respective genomes, and showed that seeds with homozygous genotypes were not viable. Additionally, in order to exclude the influence of gametophytes, reciprocal crosses were performed. The genetic transmission capacity of both females and males in the four mutants was similar to that of the wild-type (Supplementary Table S2), confirming that the gametophyte fertilities were not affected by the loss of function of *AtNSE1* or *AtNSE3*. All of these results support the conclusion that defects in *AtNSE1* and *AtNSE3* can lead to seed abortion, which may be caused by lethality of homologous embryo and endosperm.

**Table 1.** The ratio of sterile seeds in the mutant plants

Line	Nomal (%)	Sterile (%)	*n*
Wild type	98.2	1.8	934
*nse1-1/+*	74.5	25.5**	1452
*nse1-2/+*	73.2	26.8**	1007
*nse3-1/+*	74.1	25.9**	1518
*nse3-2/+*	74.1	25.9**	1347

*n*, total number of seeds examined.

** Significantly different from wild-type (*P*<0.01).

**Table 2.** Segregation rates in the AtNSE1 and AtNSE3 mutants

Cross (female×male)^a^	With T-DNA insertion (W)	Without T-DNA insertion (WO)	W:WO rate^b^	Expected rate
*nse1-1/+* × *nse1-1/+*	858	419	2.05:1^C^	3:1
*nse1-2/+* × *nse1-2/+*	330	154	2.14:1^C^	3:1
*nse3-1/+* × *nse3-1/+*	846	414	2.04:1^C^	3:1
*nse3-2/+* × *nse3-2/+*	858	402	2.13:1^C^	3:1

a Seeds obtained by each cross were grown on selective plates to determine the segregation for *nse1-1/+*, *nse3-1/+*, and *nse3-2/+*, while for *nse1-2/+*, seeds obtained by each cross were sown on non-selective plates and the segregation determined by PCR.

b With T-DNA insertion (W):without T-DNA insertion (WO).

c Significantly difference from the segregation ratio of 3:1 (*P*<0.01).

**Fig. 2.**
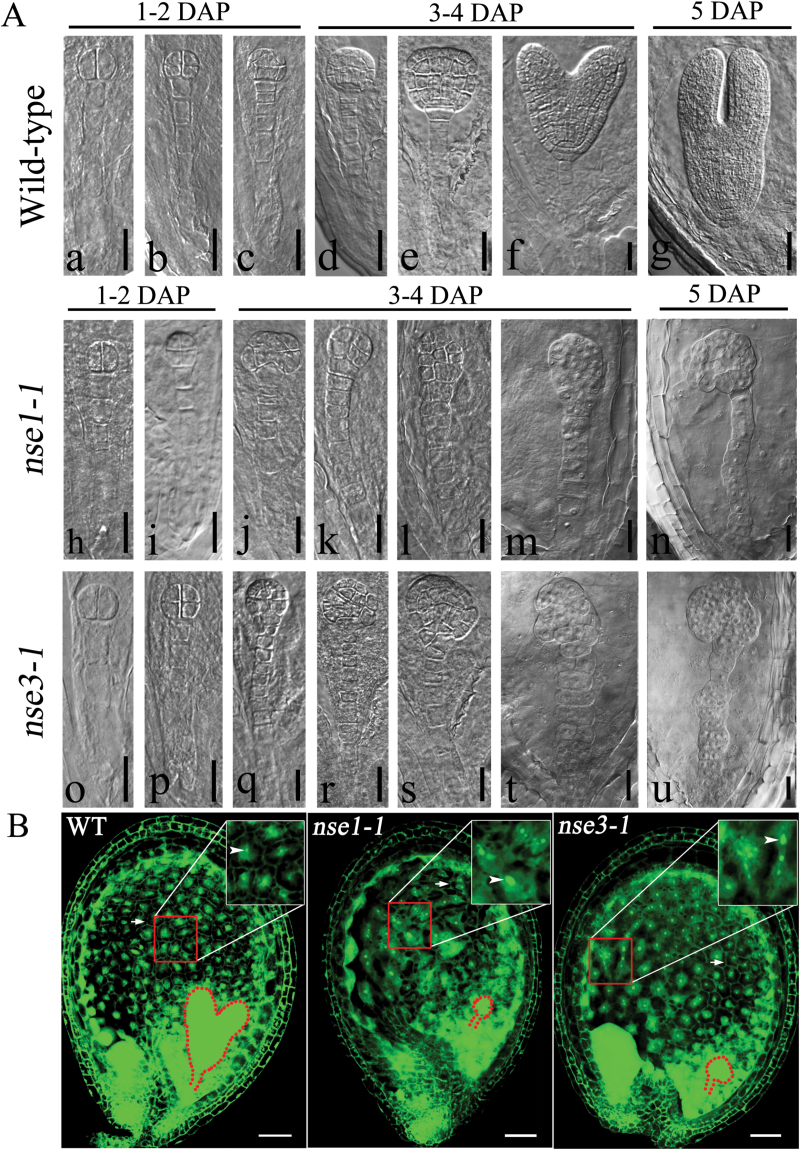
The *AtNSE1* and *AtNSE3* mutants display abnormal development in embryos and endosperms. (A) Different developmental stages of embryos in wild-type (a–g), *nse1-1* (h–n) and *nse3-1* (o–u). Abnormal embryo development occurred after 2 DAP in *nse1-1* (j–n) and *nse3-1* (q–u). Bars: 25 μm (a, b, f, h–l, o–s), 20 μm (m, n, t, u), and 50 μm (c–e, g). DAP, days after pollination. (B) Malformation of endosperm in *nse1-1* and *nse3-1* mutants. The white boxes at upper right are an enlargement of the red boxes. There are many bigger endosperm nuclei with irregular shape in *nse1-1* and *nse3-1* mutants than in wild-type (shown with arrowheads), but the cell walls of the endosperm are formed normally in the mutants (shown with arrows). WT, wild-type. Bars: 50 μm.

The embryo and endosperm are the major components of young seeds in Arabidopsis. However, it is not clear what the key factor is in the *AtNSE1* and *AtNSE3* mutants that ultimately causes seed abortion. To clarify this question, we monitored the process of embryo and endosperm development using clearing ovules and observing autofluorescence of the endosperm. One to two days after pollination, we could not distinguish between normal and abortive ovules (Fig. 2Aa–c, h, i, o, p; the data of *nse1-2* and *nse3-2* are not shown). As we know, the suspensor of wild-type often contains six to eight cells. However, beginning at 3 DAP, the 16/32 cells of the embryo proper started to divide abnormally (Fig. 2Aj–m, q–t), and the suspensor also displayed abnormal transverse and longitudinal divisions, especially after 5 DAP (Fig. 2An, u). Finally, the cell number of the mutant suspensors was more than that of the wild-type suspensor, and the abnormal division of embryos became increasingly serious as developmental time increased. Eventually, even the boundary between the embryo proper and the suspensor was indistinguishable (Fig. 2Am, t). We also observed endosperm development in *AtNSE1* and *AtNSE3* mutants by monitoring the autofluorescence of the endosperm, and found that although the cellularization process of the endosperm could be completed, the shape of the endosperm nuclei was irregular, and the size of endosperm nuclei was not uniform compared with the wild-type (Fig. 2B). This phenomenon indicated that endosperm development was defective, suggesting that the genomic DNA ploidy of the endosperm nuclei was affected to at least some extent. To confirm that the observed phenotypes were caused by the loss of *AtNSE1* and *AtNSE3*, complementation of the four mutants was performed with transgenic *pAtNSE1::NSE1-GFP* and *pAtNSE3::NSE3-GFP* plants, respectively. The results showed that homozygous mutants were obtained from the progenies of the transgenic plants through screening, and their fertility had been restored (Supplementary Fig. S1A, B). Therefore, it is clear that the loss of *AtNSE1* or *AtNSE3* function not only severely affects early embryo development, but also affects endosperm development to some extent.

### Expression of *AtNSE1* and *AtNSE3* in different tissues of Arabidopsis

**Fig. 3.**
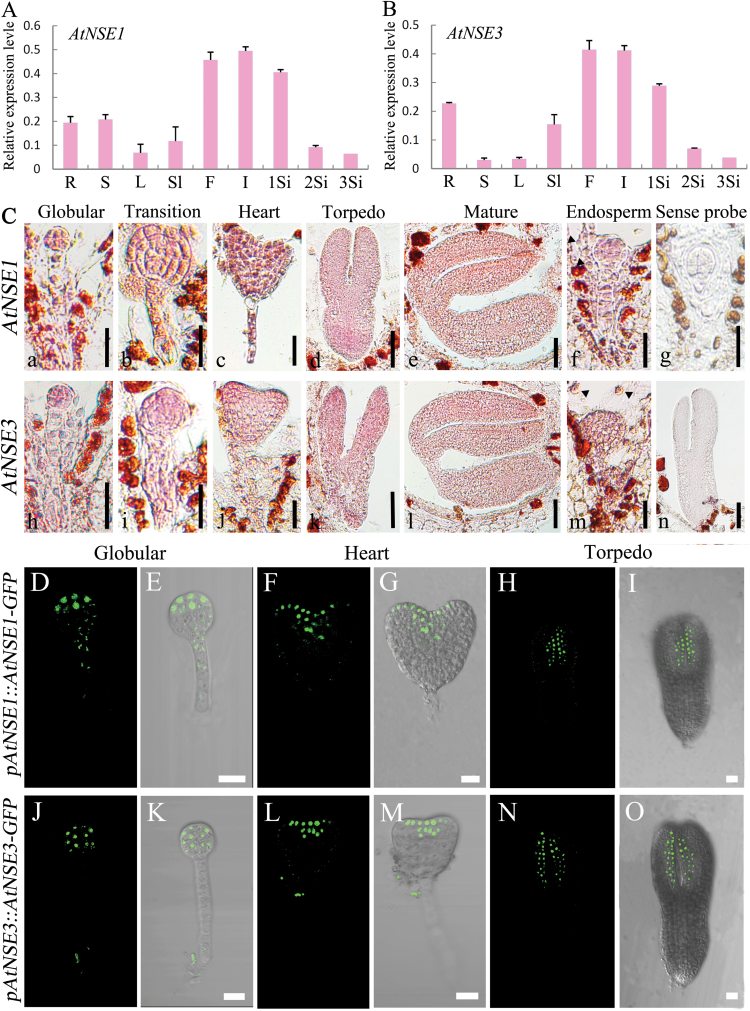
Expression assays of *AtNSE1* and *AtNSE3* genes. (A, B) Assays of temporal and spatial transcript levels of *AtNSE1* and *AtNSE3* genes with qRT-PCR in wild-type plant. R, root; S, stem; L, leaf; Sl, seedling; F, flower; I, inflorescence; 1Si–3Si, siliques at 1, 2 and 3 DAP. (C) *In situ* hybridization of *AtNSE1* and *AtNSE3* transcripts in wild-type embryos (a–e for *NSE1*, h–l for *NSE3*) and endosperm (f for *NSE1*, m for *NSE3*). Sense probes are shown in (g, n). Arrowheads show the endosperm nuclei in the ovules. Bars: 40 μm (a–c, f–k, m, n) and 50 μm (d, e, l). (D–O) Expression of *pAtNSE1::AtNSE1-GFP* and *pAtNSE3::AtNSE3-GFP* in different stages of embryos. The expression pattern of AtNSE1 is similar to AtNSE3. AtNSE1 is expressed in upper cells of globular embryo proper (D, E), and AtNSE3 in the whole globular embryo proper (J, K). They were expressed in shoot apical meristem and the primordia of the cotyledon at the stage of heart embryo (F, G, L, M) and the adaxial surface of the cotyledon at the stage of torpedo embryo (H, I, N, O) in Arabidopsis. Bars: 20 μm.

To characterize the temporal and spatial expression patterns of *AtNSE1* and *AtNSE3* in Arabidopsis, we measured their expression in many kinds of tissues using quantitative real-time PCR (qRT-PCR) assays. *AtNSE1* and *AtNSE3* had similar expression patterns and were expressed in almost all of the vegetative and reproductive tissues tested, with the highest expression detected in inflorescences (Fig. 3A, B). We also confirmed the expression patterns of the two genes in embryos and the endosperm using RNA *in situ* hybridization (Fig. 3C). Further, the expression of *pAtNSE1::NSE1-GFP* and *pAtNSE3::NSE3-GFP* more clearly displayed similar patterns to AtNSE1 (Fig. 3D–I) and AtNSE3 (Fig. 3J–O) in the embryos. Both of them were expressed throughout the process of embryo development from globular stage to torpedo stage, and more accumulated in the SAM and the primordia of cotyledon at the late stage. Thus, both *AtNSE1* and *AtNSE3* were expressed widely in Arabidopsis and had similar expression patterns, which was consistent with AtNSE1 being able to interact with AtNSE3 and their working together as components of a complex.

### AtNSE1 and AtNSE3 are conserved nuclear proteins that can directly interact with each other

Previous studies have shown that NSE1 and NSE3 play fundamental roles in cell division in yeast, and these two proteins are conserved in different species (McDonald *et al.*, 2003; Pebernard *et al.*, 2004, 2008; Doyle *et al.*, 2010; Tapia-Alveal and O’Connell, 2011; Hudson *et al.*, 2011; Taniura *et al.*, 2015; Palecek and Gruber, 2015). In Arabidopsis, the orthologues of *NSE1* and *NSE3* have been predicted to be *AtNSE1* (At5g21140, AtEMB1379) and *AtNSE3* (At1g34770) (Losada and Hirano, 2005). Here, we performed alignments of the predicted amino acid sequences of these proteins from highly diverse species, including animals, plants, and yeast, and modeled their three-dimensional structures. The homology of AtNSE1 and AtNSE3 in different species was not high at the primary structure level (Supplementary Fig. S2A, B), but both proteins had conserved functional domains at the tertiary structure level, including two winged-helix domains (WHDs) and a RING-like domain in AtNSE1 (Supplementary Fig. S2C, D) and a MAGE domain (including WHA and WHB) in AtNSE3 (Supplementary Fig. S2C, E). These conserved domains suggest that both AtSE1 and AtNSE3 may have conserved functions in Arabidopsis.

**Fig. 4.**
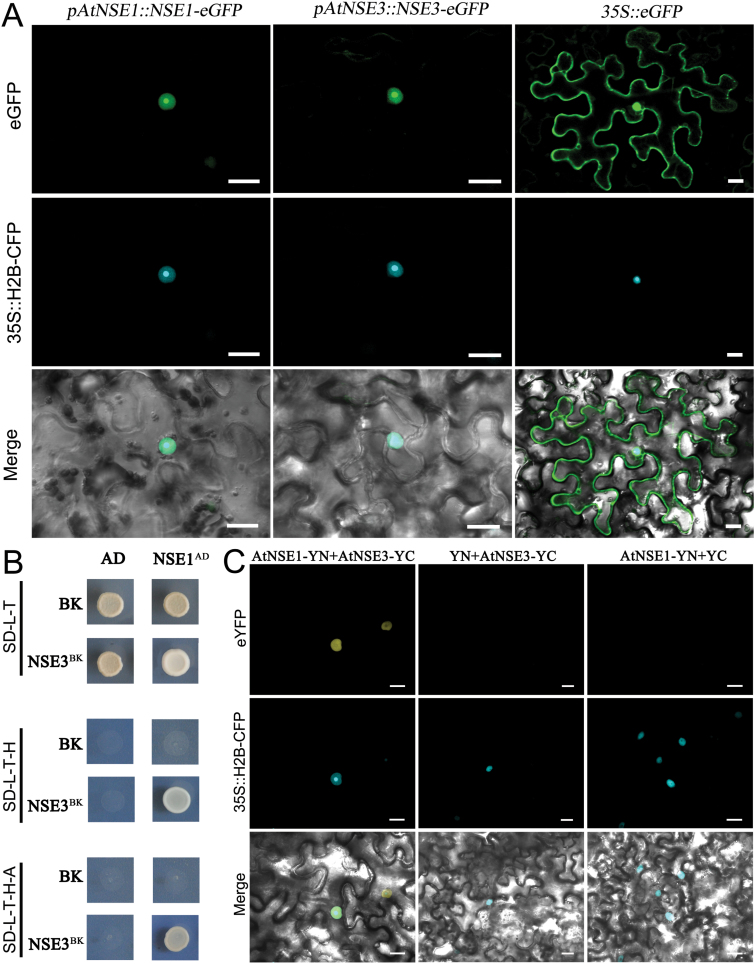
AtNSE1 and AtNSE3 are nuclear proteins and can interact with each other. (A) Transient expression of AtNSE1 and AtNSE3 in tobacco showing they are localized in nuclei. *35S::eGFP* is the control. Bars: 20 μm. (B) AtNSE1 and AtNSE3 can interact with each other in yeast. Yeast two-hybrid assays were performed and the co-transformed strains were spotted on SD–L–T/SD–L–T–H/SD–L–T–H–A plates to test the direct interaction between the expressed proteins. AD, pGADT7 vector; BK, pGBKT7 vector; SD, synthetic dextrose; L, Leu; T, Trp; H, His; A, adenine. (C) AtNSE1 and AtNSE3 can interact in tobacco leaf epidermis cells. The epidermal cells were observed at 2 d after being co-transformed. YC, YFP C-terminal fragment (aa156-239); YN, YFP N-terminal fragment (aa 1–155). Bars: 20 μm. The 35S::H2B-CFP is a nuclear marker.

It has been demonstrated that both NSE1 and NSE3 are nuclear proteins, and that both proteins can interact with each other in yeast (Sergeant *et al.*, 2005; Pebernard *et al.*, 2008; Doyle *et al.*, 2010; Kozakova *et al.*, 2015). Interestingly, a recent study of the amoeba *Dictyostelium discoideum* showed that NSE1 was expressed extensively in cells, and also found that when NSE1 was co-expressed with NSE3, it was readily translocated to the nucleus (Taniura *et al.*, 2015). In our experiments, we initially evaluated AtNSE1 and AtNSE3 localization in *Nicotiana benthamiana* using *pNSE1::NSE1-GFP* and *pNSE3::NSE3-GFP* constructs for transient transformation, and observed that their green fluorescent protein (GFP) signals were localized specifically in nuclei (Fig. 4A). We subsequently used yeast two-hybrid assays to investigate the potential interactions of these proteins, and found that yeast cells co-expressing AtNSE1 as bait and AtNSE3 as prey (Fig. 4B), or cells co-expressing AtNSE3 as bait and AtNSE1 as prey (data not shown), could grow on SD/–His–Leu–Trp and SD/–His–Leu–Trp–Ade media, indicating that AtNSE1 and AtNSE3 very readily interact with each other on high-stringency selection plates. To further confirm the interaction between AtNSE1 and AtNSE3, we also performed a BiFC assay in leaves of *Nicotiana* using transient transformation. The yellow fluorescent protein (YFP) signal was only observed in transformed leaf cells co-expressing the constructs *AtNSE1-YN* and *AtNSE3-YC* (Fig. 4C) or *AtNSE1-YC* and *AtNSE3-YN* (data not shown). Moreover, the YFP fluorescence signal accumulated exclusively and obviously in the nuclei, thereby establishing an additional level of evidence confirming the GFP assay results showing the subcellular localization of AtNSE1 and AtNSE3. Together, these results show that both AtNSE1 and AtNSE3 are conserved nuclear proteins that can interact with each other directly, implying that these proteins may work together as a complex in Arabidopsis.

### Transcriptome analysis of the *nse1-1* and *nse3-1* mutants

To further investigate the effects of mutations in *AtNSE1* and *AtNSE3* on gene expression, we performed transcriptome analysis of homozygous abortive white ovules from *nse1-1/+* and *nse3-1/+* plants. The Venn diagrams of differentially expressed genes (at least a two-fold difference in expression) indicated that 2064 genes had up-regulated expression in *nse1-1* and that 1655 genes had up-regulated expression in *nse3-1*; 2863 genes in *nse1-1* and 2633 genes in *nse3-1* had down-regulated expression. Interestingly, among these genes, most of them were differentially expressed in both *nse1-1* and *nse3-1* ovules (1440 up-regulated genes and 2428 down-regulated genes shared by both) (Supplementary Fig. S3A). This result is consistent with the notion that AtNSE1 and AtNSE3 work together as a complex. To explore which biological progresses were influenced by mutations in *AtNSE1* and *AtNSE3*, we performed analysis of gene ontology (GO) classification based on RNA-seq data. Various genes involved in cell cycle regulation and responses to DNA damage stimulus were up-regulated while other genes involved in cell fate, cell differentiation, and organ and tissue development were down-regulated (Supplementary Fig. S3B). Further, Kyoto Encyclopedia of Genes and Genomes (KEGG) pathway enrichment analysis of the differentially expressed genes showed that the DNA replication, mismatch repair pathway, base excision pathway and homologous recombination (HR) pathway were significantly activated (Supplementary Fig. S3C). All of these results indicated that *AtNSE1* and *AtNSE3* may function in cell division, tissue and organ morphogenesis, and DNA damage repair.

### The cell division activity is reduced in the partially complemented mutant seedlings

To determine whether or not *AtNSE1* and/or *AtNSE3* functions during post-embryonic development, we performed partial complementation experiments using the seed-specific *ABI3* promoter to drive the expression of fusions of GFP to the coding sequence of *AtNSE1* or *AtNSE3* (*pABI3::AtNSE1-GFP*, *pABI3::AtNSE3-GFP*). Through resistance screening, we obtained partially complemented *nse1-1/+ ABI3::NSE1* and *nse3-1/+ ABI3::NSE3* plants. We first calculated the ratio of aborted ovules in the progenies of these plants. Only 6.23% (*n*=1108) and 1.38% (*n*=1063) aborted ovules were observed in the *nse1-1/+ ABI3::NSE1* and *nse3-1/+ ABI3::NSE3* plants, respectively, clearly many fewer aborted ovules than were observed in *nse1-1/+* (25.5%) and *nse3-1/+* (25.9%) plants. These results indicated that the *ABI3* promoter could drive *AtNSE1* and *AtNSE3* to partially complement the defects in embryo development present in *nse1-1* and *nse3-1*.

**Fig. 5.**
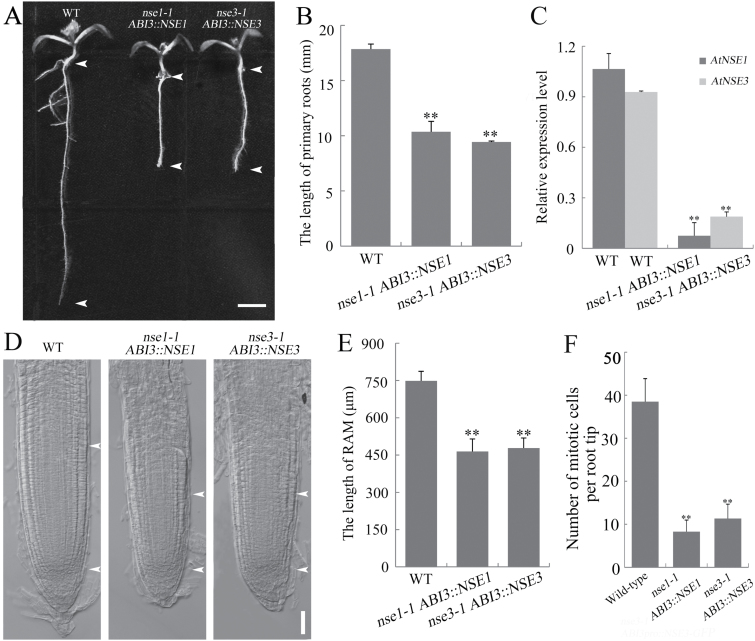
Partially complemented mutants show inhibited growth. (A) The primary root of the mutants is shorter than that of wild-type. The seedlings were grown on 1/2 MS medium for 1 week. The distance between the arrowheads shows the length of primary root. Bars: 3 mm. (B) Analysis of primary root length. (C) Relative expressions of *AtNSE1* and *AtNSE3* in 2-week-old seedlings of wild-type, *nse1-1 ABI3::NSE1-GFP* and *nse3-1 ABI3::NSE3-GFP*. (D) The root apical meristem of the mutants is shorter than that of wild-type. Primary root of seedlings germinated after 5 d. The distance between the arrowheads shows the length of root apical meristem (RAM) of primary root. Bars: 50 μm. (E) Analysis of RAM length. (F) The number of mitotic cells per root tip is decreased in the mutant seedlings. Seedlings at 5 DAG of wild-type, *nse1-1 ABI3::NSE1* and *nse3-1 ABI3::NSE3* were used. **Significant difference according to Student’s *t*-test (*P*<0.01).

Of note, a portion of the abnormal seedlings were obtained from the progenies of *nse1-1*/+ *ABI3::NSE1* and *nse3-1*/+ *ABI3::NSE3* (with 14.7%, *n*=374 and 17.7%, *n*=401, respectively), and they were homozygous at each gene locus as confirmed by PCR assay (Supplementary Fig. S1A). And we found that the primary root of the mutant seedlings was shorter significantly than in the wild-type (Fig. 5A). We measured the lengths of primary roots in 5-day-old seedlings. The average length of the primary root of *nse1-1*/- *ABI3::NSE1* and *nse3-1*/- *ABI3::NSE3* seedlings was 41.96% and 47.13% shorter, respectively, than in the wild-type seedlings (Fig. 5B). We also analysed the expression of *NSE1* and *NSE3* in *nse1-1*/- *ABI3::NSE1* and *nse3-1*/- *ABI3::NSE3* seedlings at 2 weeks, and found that both of them had significantly down-regulated expression (Fig. 5C). In 10-day-old mutant seedlings, we also found that *NSE1* and *NSE3* were down-regulated significantly in root and shoot (Supplementary Fig. S4A, B). Further, we measured lengths of root apical meristem (RAM) in 5-day-old seedlings. The average length of the RAM of the *nse1-1*/- *ABI3::NSE1* and *nse3-1*/- *ABI3::NSE3* seedlings was 37.98% and 36.10% shorter than wild-type seedlings, respectively (Fig. 5D, E). Together these results implied that the cell division activity may be inhibited. To confirm that, we evaluated the mitotic activity in the RAM of partially complemented seedlings. In contrast to wild-type seedlings, the number of dividing cells in the RAM was reduced in the partially complemented plants (8.29 in *nse1-1/+ ABI3::NSE1*, 11.3 in *nse3-1/+ ABI3::NSE3*, and 38.5 in wild-type on average) (Fig. 5F), showing that the mitotic activity was indeed decreased significantly. In addition, in the partially complemented mutants, the second pair of leaves could not be differentiated from the meristems (Supplementary Fig. S1C). About 2 weeks after seed germination, the *nse1-1*/- *ABI3::NSE1* and *nse3-1*/- *ABI3::NSE3* seedlings did not continue development and eventually died. These results suggested that the maintenance of the shoot apical meristem (SAM) was out of control in the partially complemented mutant plants. This implies that malfunction of *AtNSE1* and *AtNSE3* can regulate the growth of seedlings via interference in shoot meristem activity. We used RNA-seq to examine some of the genes known to be involved in RAM and SAM maintenance, including *WOX5*, *TMO7*, *PLT1*, *CLV1*, and *CLV3* in 2-week-old wild-type, *nse1-1 ABI3::NSE1*, and *nse3-1 ABI3::NSE3* seedlings. Compared with wild-type seedlings, all of these genes had down-regulated expression in the partially complemented seedlings (Supplementary Fig. S4C), suggesting that *AtNSE1* and *AtNSE3* are involved in RAM and SAM activity maintenance at the RNA-transcriptional level.

### Mitotic division was inhibited in partially complemented homozygous *nse1-1* and *nse3-1* mutant plants

**Fig. 6.**
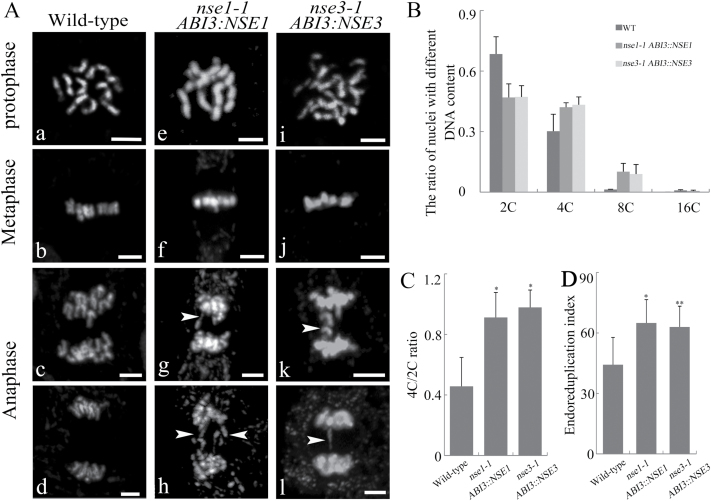
Mutations of *AtNSE1* and *AtNSE3* induce defects of mitosis and facilitate endoreduplication. (A) Chromatid segregation shows abnormality during anaphase. The root tip cells were detected in 5 DAG seedlings of wild-type (a–d), *nse1-1 ABI3::NSE1* (e–h), and *nse3-1 ABI3::NSE3* (i–l). Arrowheads show lagging chromosomes and chromosome bridges. Bars: 10 μm. (B–D) The ratios of nuclei with different DNA content (B), the 4c/2c ratio (C) and endoreduplication (D) of true leaves’ nuclei in wild-type, *nse1-1 ABI3::NSE1*, and *nse3-1 ABI3::NSE3* seedlings at 10 days after germination. The asterisks indicate a statistically significant difference according to Student’s *t*-test (*P<0.05, **P<0.01).

The early mutant embryos displayed abnormal cell division and reduced mitotic activity, which was the same as the mutant seedlings. We monitored the process of mitosis in the root tip cells of wild-type and mutant seedlings. There were no differences between the wild-type and the mutants until anaphase (Fig. 6Aa, b, e, f, i, j). In the anaphase cells of the mutants, we observed that some cells contained lagging chromosomes (Fig. 6Ag, k) and chromosomal bridges (Fig. 6Ah, l) (61.33% in *nse1-1 ABI3:NSE1*, *n*=75; 44.04% in *nse3-1 ABI3::NSE3*, *n*=193; none in wild-type, *n*=428), indicating that the chromosome segregation in anaphase during mitosis was hindered due to mutations in *AtNSE1* and *AtNSE3*. We used flow cytometry assays to further analyse the cell cycle in the first pair of leaves from 10-day-old wild-type and partially complemented mutant seedlings. The number of 4C nuclei was larger in the mutants than in the wild-type (Fig. 6B). The proportion of 4C to 2C nuclei was also much higher in the mutants (Fig. 6C), indicating that more nuclei had undergone DNA replication but did not undergo mitosis, and the cell cycle was delayed at the G2/M phase. The endoreduplication index was also higher in the mutants than in the wild-type seedlings (Fig. 6D). Therefore, the loss of function of *AtNSE1* or *AtNSE3* led to G2/M delay and endoreduplication in the mutants, which may be a reason why the mitosis activity of the mutant RAM was decreased and the seedling growth was inhibited. At the same time, this result was consistent with the non-uniform nuclear size phenotype that was observed in the endosperm of *nse1-1* and *nse3-1* embryo sacs. *KRP6* is an important regulator of the cell cycle. Overexpression of *AtKRP6* leads to multinucleated cells in Arabidopsis cell cultures and root cells (Vieira *et al.*, 2014). In both our RNA-seq and qRT-PCR data, expression of *AtKRP6* was significantly up-regulated in the partially complemented mutant seedlings as compared with the wild-type seedlings (Supplementary Fig. S4C). These results show that both *AtNSE1* and *AtNSE3* are crucial factors for maintaining cell division and preserving diploidy in somatic cells.

### *AtNSE1* and *AtNSE3* are required for DNA DSBs repair in somatic cells

**Fig. 7.**
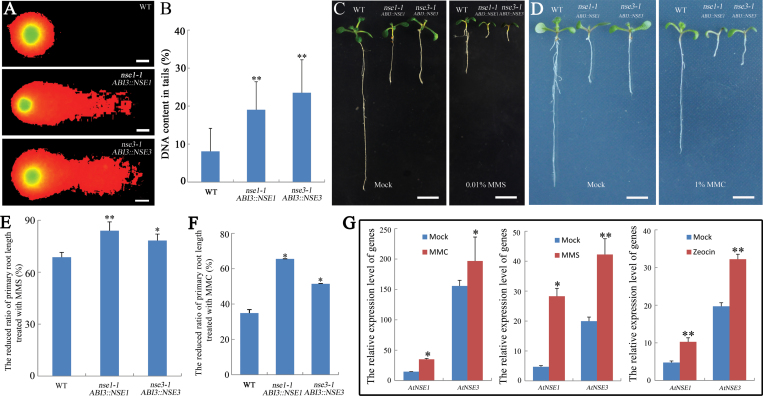
*AtNSE1* and *AtNSE3* are involved in DNA damage repair. (A) DSBs are increased in the mutant seedlings. Commet assay in wild-type, *nse1-1 ABI3::NSE1*, and *nse3-1 ABI3:NSE3* seedlings. Bars: 10 μm. (B) DNA damage as measured in commet assay, showing the percentage of DNA content in the tail of nuclei for WT, *nse1-1 ABI3::NSE1*, and *nse3-1 ABI3:NSE3* seedlings. The mean value of more than 100 nuclei is shown with SD bars. (C–F) The mutants are more sensitive to MMS and MMC. Ten-day-old seedlings of wild-type, *nse1-1 ABI3::NSE1*, and *nse3-1 ABI3:NSE3* were treated on 1/2 MS plates containing 0.01% MMS and 1% MMC (mg 100 ml^–1^), and statistical analysis was performed for the reduced ratio of primary roots length. The reduced ratio=(length of WT with no MMS or MMC–length of mutant with MMS or MMC)/length of WT with no MMS or MMC. Bars: 0.5 cm. (G) The relative expression levels of *AtNSE1* and *AtNSE3* response to MMC, MMS, and Zeocin in suspension cells. The asterisks indicate a statistically significant difference according to Student’s *t*-test (*P<0.05, **P<0.01).

Our RNA-seq analysis indicated that *AtNSE1* and *AtNSE3* may be involved in homologous recombination-mediated DSB repair (Supplementary Fig. S3C). We used qRT-PCR to verify the RNA-seq expression results for several genes involved in DNA repair in the *nse1-1 ABI3::NSE1* and *nse3-1 ABI3::NSE3* seedlings. Although *KU70* and *KU80*, which are known to participate in the non-homologous end joining (NHEJ) pathway, had no changes at the transcriptional level in the mutants, some genes involved in HR-mediated DSB repair, including *Rad51*, *POLD4*, *RPA1e*, *and RPA70c*, were significantly up-regulated in the mutants (Supplementary Fig. S4C). These results suggested that the mutations of *AtNSE1* and *AtNSE3* may cause DSB repair defects. The commet assays showed that the amount of DSBs was obviously increased in the *nse1-1 ABI3::NSE1* and *nse3-1 ABI3::NSE3* seedlings as compared with the wild-type seedlings (Fig. 7A, B). At the same time, using propidium iodide (PI) staining, we observed that the dead cells also increased in the root tips of 5-day-old partially complemented seedlings (Supplementary Fig. S5). Further, after being treated by 0.01% methyl methanesulphonate (MMS; a DNA cross-linking agent which can cause DSBs) (Xu *et al.*, 2013; Wang *et al.*, 2014), the length of the mutant seedlings’ primary roots was significantly reduced compared with the wild-type seedlings (68.65% in wild-type, 83.96% in *nse1-1 ABI3::NSE1* and 78.30% in *nse3-1 ABI3::NSE3*) (Fig. 7C, E), indicating that the *AtNSE1* and *AtNSE3* mutants were more sensitive to MMS than was the wild-type. For another DNA cross-linking agent, mitomycin C (MMC; 1%; Wang *et al.*, 2014), similar results were obtained (Fig. 7D, F). In addition, we analysed the expression of *AtNSE1* and *AtNSE3* in Arabidopsis suspension cells that were treated with different DNA damage-inducing agents. The expression of these two genes was up-regulated in all MMS, MMC, and Zeocin (DSB inducer) treated suspension cells (Fig. 7G). These results suggest that both *AtNSE1* and *AtNSE3* are involved in DSB repair. *AtSOG1* (suppressor of gamma response 1) is a specific transcription factor in Arabidopsis that is known to be involved in responses to DNA damage (Yoshiyama *et al.*, 2009, 2013, 2014). We analysed the expression of *AtNSE1* and *AtNSE3* in the transgenic plants overexpressing *AtSOG1* and found that both *AtNSE1* and *AtNSE3* were up-regulated significantly (Supplementary Fig. S4D), suggesting that the expression of *AtNSE1* and *AtNSE3* may be associated with *AtSOG1*, to some extent, in response to the DNA damage repair pathway.

As a result, we suggest that the mutation of *AtNSE1* and *AtNSE3* led to the situation that the DSBs, which were induced during mitosis, could not be repaired efficiently. These DSBs accumulated in the somatic cells, and induced cell cycle delay and occurrence of endoreduplication, which finally led to reduced mitotic activity and instability of the genome. Thus, the mutant embryos and seedlings displayed abnormal cell divisions and inhibited growth, and finally lethality.

## Discussion

### *AtNSE1* and *AtNSE3* are crucial factors for maintaining embryo and post-embryonic development in Arabidopsis

In yeast, previous studies have shown that NSE1 and NSE3 are important components of the SMC5/6 complex. Both proteins can interact with NSE4 to support the function of the SMC5/6 complex, and knock-out mutants lacking these proteins are lethal (Pebernard *et al.*, 2008; Guerineau *et al.*, 2012; Kozakova *et al.*, 2015; Verver *et al.*, 2016). Recently, it was reported that the SMC5/6 complex is involved in crucial steps during human spermatogenesis (Verver *et al.*, 2014). Complete knockout of *Smc6* in mice resulted in early embryonic lethality, demonstrating that this gene is essential for embryo development in mammals (Ju *et al.*, 2013). In Arabidopsis, *AtMMS21/NSE2* is involved in embryo development and the maintenance of the root stem cell niche. Mutation of *AtMMS21/NSE2* results in cell death in Arabidopsis roots (Xu *et al.*, 2013). Mutation of *AtSMC5* led to embryo lethality (Watanabe *et al.*, 2009; Xu *et al.*, 2013). However, there were still no characterizations of the roles of *AtNSE1*, *AtNSE3*, and *AtNSE4A/4B* in plant development. To understand their functions in the SMC5/6 complex in plant development, we investigated the phenotypes of their mutants, and found that the homologous mutation of *AtNSE1* or *AtNSE3* was lethal. However, we did not obtain knock-out mutants of *AtNSE4A* and *AtNSE4B*. In yeast, NSE4, with NSE1 and NSE3 together, constituted an important subcomplex bridging the head of SMC5 and SMC6, and the *nse4*^*ts*^ mutant was hypersensitive to DNA damage (Hu *et al.*, 2005; Palecek *et al.*, 2006). In Arabidopsis, NSE4 was encoded by two homologous genes, *AtNSE4A* and *AtNSE4B*. Whether these two genes had new functions in Arabidopsis was not quite clear. In this study, we focused on the functions of AtNSE1 and AtNSE3, and found that they are conserved nuclear proteins that can interact with each other. Mutations in *AtNSE1* and *AtNSE3* led to disordered cell mitosis in early embryo development, finally resulting in sterile seeds in Arabidopsis. Thus, we believe that the SMC5/6 complex plays essential roles in embryo development whether in animals or in plants. And all the subunits of SMC5/6 could be indispensable during this process although the roles of *AtNSE4A* and *AtNSE4B* have not been revealed in Arabidopsis. Our study showed that, different from *AtMMS21/NSE2*, *AtASAP1/NSE5*, and *AtSNI1/NSE6* for which homozygous plants could be obtained, the *AtNSE1* and *AtNSE3* homologous mutants were lethal and had more severe phenotypes in embryo development compared with the other mutants (Fig. 2). The T-DNA insertion of *mms21-1* was located at the intron adjacent to the last exon (Huang *et al.*, 2009), and it may be a weak mutation in Arabidopsis. The mutations of *AtASAP1* and *AtSNI1* were point mutations (Yan *et al.*, 2013). This might be a reason why they were not lethal and displayed a different phenotype compared with *nse1-1* and *nse3-1*, although they were all components of the SMC5/6 complex.

Post-embryonic development is essential for plant individual development (Xing *et al.*, 2008). Post-embryonic formation of organs initially arises from shoot and root apical meristems (SAM and RAM) (Besnard *et al.*, 2014; Sozzani and Iyer-Pascuzzi, 2014). Thus, maintenance of these meristems is very important for post-embryonic development. It was reported that many transcription factors were involved in maintenance of SAM and RAM. *CLV1* and *CLV3* played an essential role in the *WUS-CLV* signaling pathway, which is required for regulation of SAM (Fletcher *et al.*, 1999; Nimchuk *et al.*, 2011; Bustamante *et al.*, 2016), while *WOX5*, *TMO7* and *PLT1* were effective in RAM maintenance (Tian *et al.*, 2014; Forzani *et al.*, 2014; Aida *et al.*, 2004; Schlereth *et al.*, 2010). The analysis of the partially complemented mutants showed that the mutations of *AtNSE1* and *AtNSE3* led to arrested seedling growth and eventual plant death (Fig. 5). Moreover, organ differentiation, especially for SAM and RAM (Fig. 5, Supplementary Fig. S1), was also suppressed. Several genes known to be involved in cell fate and organ formation were significantly down-regulated in mutant ovules and seedlings (Supplementary Figs S3 and S4). Based on these results, we conclude that *AtNSE1* and *AtNSE3* are required for seedling growth and differentiation of shoot and root meristem tissues and these genes may start to function at the early embryogenesis stage.

### *AtNSE1* and *AtNSE3* are involved in DSB repair and maintaining the stability of chromosome ploidy in mitosis

Studies in yeast and mammals have verified that SMC5/6 is a very complicated protein complex. It is involved not only in the regulation of mitosis but also in meiosis, having roles in restarting stalled replicated forks, in homologous recombination, in the maintenance of heterochromatin and ribosomal DNA, and in the regulation of chromosome topology and telomerase-independent telomere elongation (Verver *et al.*, 2016). In plant development, the functions of the SMC5/6 complex have not yet been elucidated. It has been noted that *AtMMS21/NSE2* is involved not only in DSB repair in somatic cells but also in gametophyte development (Xu *et al.*, 2013; Liu *et al.*, 2014*a*). *AtSMC6A* and *AtSMC6B* also play important roles in DSB repair via the HR repair pathway (Watanabe *et al.*, 2009; Liu *et al.*, 2015). ASAP1 and SNI1 were identified as NSE5 and NSE6 in Arabidopsis, and the research indicated that SMC5/6 negatively regulates RAD17 and ATR (Yan *et al.*, 2013). In our study, we found that the mutant of *AtNSE1* and *AtNSE3* displayed a series of DNA damage responses. The HR pathway was activated in the *nse1* and *nse3* mutants (Supplementary Figs S3 and S4). All the results indicated that, as a component of the AtSMC5/6 complex, the proteins encoded by *AtNSE1* and *AtNSE3* may be involved in the regulation of the HR-mediated DSB repair pathway. Furthermore, we noted that other DNA damage repair pathways, including the mismatch repair pathway and base excision repair pathway, were also activated in the mutants (Supplementary Fig. S3). This result suggested that SMC5/6 might have a more extensive function in DNA repair. However, NHEJ is the major mode of DSB repair in higher eukaryotes (Puchta, 2005). In Arabidopsis, both *AtNSE1* and *AtNSE3* might be involved in HR and NHEJ as well as the other pathways, which needs to be investigated further.

It was reported that X-shaped sister chromatid junctions (SCJs) accumulate at stalled replication forks that are induced by a *NSE2* mutation in yeast (Branzei *et al.*, 2006). In the smc6 mutant, similar abnormal joint molecules (JMs) accumulated at the collapsed replication fork, correlating with chromosome missegregation. This research suggested that the SMC5/6 complex may be required for preventing the formation of replication stress-induced SCJs or for helping with their resolution. The efficient and timely resolution of recombination intermediates is essential for chromosome segregation at anaphase. When recombination intermediates are not properly resolved, aberrant JMs can emerge that have the potential to block chromosome segregation (Jessop and Lichten 2008; Copsey *et al.*, 2013; Xaver *et al.*, 2013). In addition, SMC5/6 was also required for maintenance of chromosome morphology, ensuring the proper chromosome segregation during mitosis (Carter and Sjögren, 2012; Jeppsson *et al.*, 2014; Gallego-Paez *et al.*, 2014). In our study, we found that the DNA replication pathway was activated most significantly (Supplementary Fig. S3), chromosome segregated aberrantly at anaphase (Fig. 6), and the DSBs could not be repaired efficiently in *nse1* and *nse3* mutants (Fig. 7). MMC induces DSBs when cross-link repair interferes with DNA replication, and HR might be involved in postreplication repair (Watanabe *et al.*, 2009). The partially complemented mutant seedlings were more sensitive to the DNA cross-linking agents MMC and MMS than wild-type (Fig. 7), suggesting that more DSBs occurring in the mutant seedlings inhibited the root growth. We speculate that numerous DSBs were occurring during DNA replication, and accumulated in mutant cells; then stalled replication forks were formed and finally led to missegregation and failed mitosis.

*AtSOG1* is a unique plant transcription factor that governs DNA damage responses with the help of ATM-mediated phosphorylation and may function in the cell cycle, DNA repair, programmed cell death, and endoreduplication (Yoshiyama *et al.*, 2009, 2013, 2014). Interestingly, *AtSOG1* is expressed specifically in the vascular tissues of cotyledons, in roots, in lateral root primordia, and in root tips (Yoshiyama *et al.*, 2013), and this is similar to the patterns of *AtNSE1* and *AtNSE3*. We also found that *AtNSE1* and *AtNSE3* had significantly up-regulated expression in *AtSOG1* overexpression transgenic lines (Supplementary Fig. S4). Therefore, we propose that AtNSE1 and AtNSE3 may be regulated by ATM-mediated SOG1 at the transcriptional level.

The DNA damage often affected cell cycle progression. Many genes associated with cell cycle regulation were up-regulated from our GO analysis in *nse1-1* and *nse3-1* (Supplementary Fig. S3). DNA damage can activate a check-point response, which can delay the cell cycle progression and allow time to repair the DNA damage. It is reported that the cells that contained much DNA damage tended to stop dividing and undergo endoreplication (Kirik *et al.*, 2007). Our results indicate that the cells could finish genome replication but could not make the transition from the G2 to the M phase, resulting in more polyploid cells in the mutants (Fig. 6). Consistent with this was the observation that there were many swelling and malformed nuclei in the endosperm of the mutants. We also found chromosome missegregation at anaphase (Fig. 6). So, we speculated that *AtNSE1* and *AtNSE3* may be able to affect the transition from the G2 to the M phase in the cell cycle and may suppress DNA damage-induced endoreduplication, preserving the diploidy and genome stability of somatic cells. All these data supported the notion that the activity of cell division in the mutant embryos and roots of the partially complemented mutant seedlings was inhibited and the growth of the mutant seedlings was slower. When more and more DSBs accumulated in the embryo or seedlings, many more cells would gradually die. Thus, the mutant embryos and seedlings died finally. However, whether SMC5/6 could regulate the development-associated factors and stimulate plant development directly is unknown. We hypothesize this complex might indirectly affect the expression of many DNA repair response factors and organ development regulators through regulation of chromatin structures.

*NSE1* and *NSE3* have been shown to function in DNA repair in yeast (Pebernard *et al.*, 2004, 2008), but their functions in Arabidopsis had not been revealed. Our research demonstrated that *AtNSE1* and *AtNSE3* were essential for the function of the SMC5/6 complex and for maintaining embryogenesis and post-embryonic development by facilitating DNA repair and maintaining cell cycle stability in Arabidopsis.

## Supplementary Material

Supplementary Data
