# Supplementary material for: Non-SMC elements 1 and 3 are required for early embryo and seedling development in Arabidopsis
Source: J Exp Bot. 2017 Feb 16;68(5):1039–54. doi: 10.1093/jxb/erx016 (PMC5441860; doi:10.1093/jxb/erx016)
Supplement: Supplementary Data [file erx016_Supplementary_Data.zip › Supplementary_Figures_S1_S5_Tables_S1_S2.pdf]

Supplementary Figures

Fig. S1

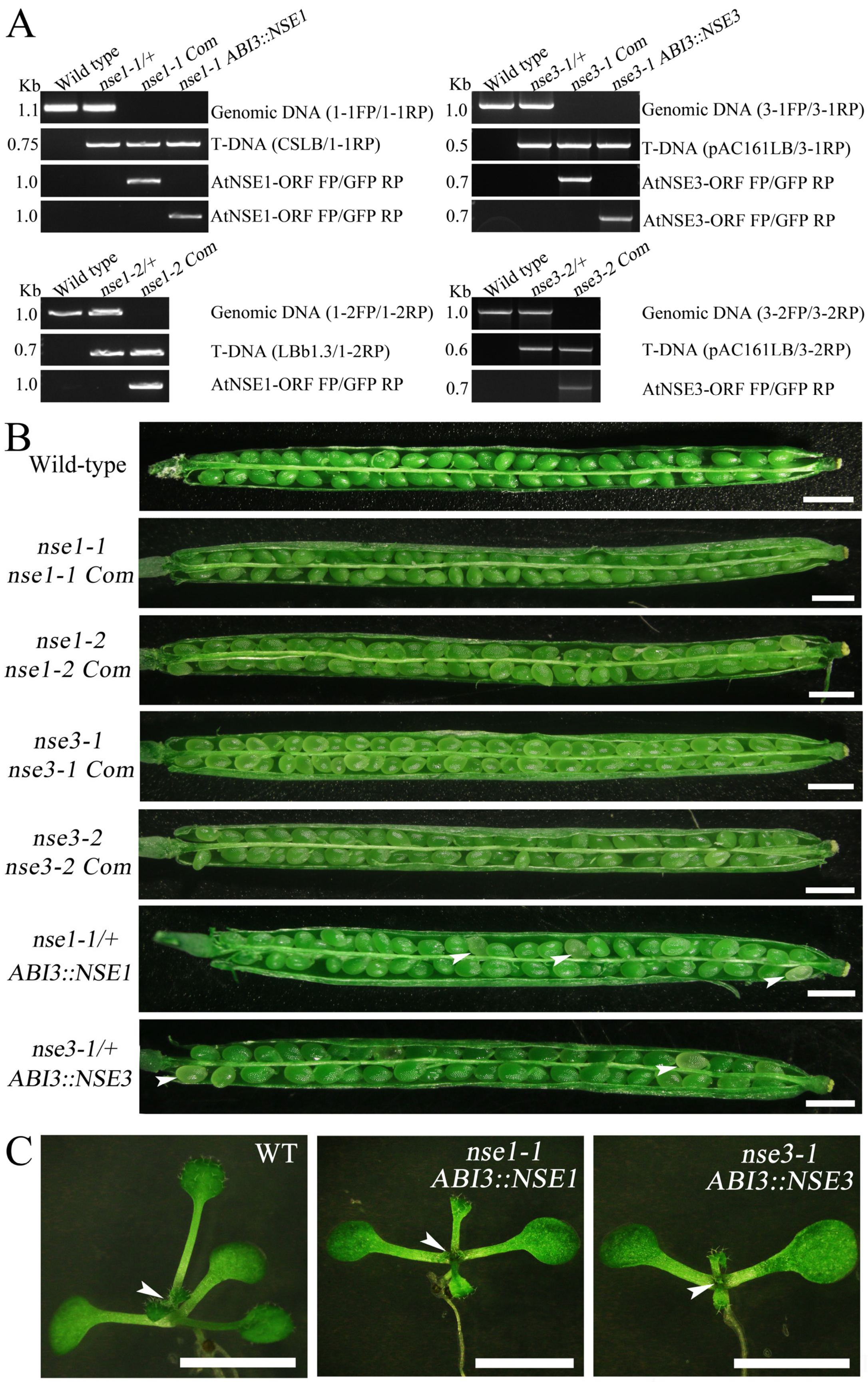

Fig. S1 Complementation phenotype and partial complementation assays of *AtNSE1* and *AtNSE3* mutants.

(A) Genotypic analysis of *nse1-1/+*, *nse1-2/+*, *nse3-1/+* and *nse3-2/+* mutants, and genotypic confirmation of the homozygous transgenic mutant plants. Kb, kilobase; FP, forward primer; RP, reverse primer; LBb1.3 and pAC161LB, primer at left border of the vector. (B) Phenotypes of the siliques in the homozygosis transformed complemented (com) plants based on the *nse1-1/+*, *nse1-2/+*, *nse3-1/+* and *nse3-2/+* mutants, and partial complemented plants based on the *nse1-1/+* and *nse3-1/+* mutants. Arrowheads show abortive white ovules. Bars=1 mm. (C) The growth of true leaves is severely inhibited in the mutant seedlings. The seedlings of WT, *nse1-1 ABI3::NSE1-GFP* and *nse3-1 ABI3::NSE3-GFP* grown on 1/2 MS medium for two weeks. Arrowheads show shoot apical. Bars=5 mm.

Fig. S2

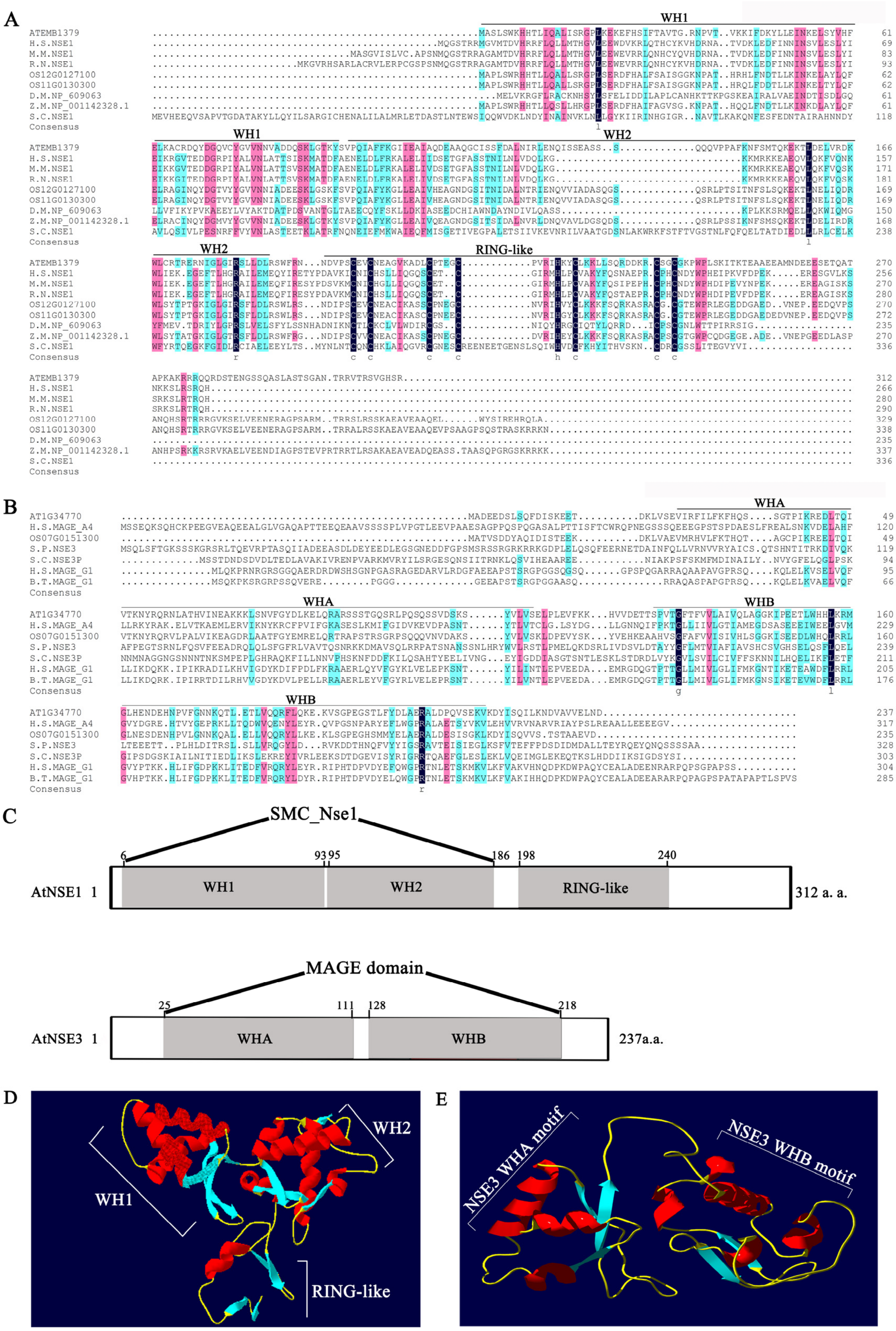

Fig. S2 The sequence alignment of homologous proteins and conserved domain of AtNSE1 and AtNSE3.

(A-B) Sequence alignment of homologous proteins in different species. Identical residues occurring in all organisms are indicated in deep blue and the similar residues are indicated in pink and baby blue. At, *Arabidopsis thaliana*; HS, *Homo sapiens*; Mm, *Mus musculus*; Rn, *Rattus norvegicus*; Dm, *Dorsophila melanogaster*; Zm, *Zea mays*; Os, *Oryza sativa*; Sc, *Saccharomyces cerevisiae*, ; Bt, *Bos taurus*; Sp, *Schizosaccharomyces pombe*. The National Center for Biotechnology Information accession number for NSE1 showed in this study and the supporting information are At5g21140 (AtNSE1, GI: 30688163),

HSNSE1 (GI: 94721324), MmNSE1 (GI: 34328291), RnNSE1 (GI: 88853863), Os12g0127100 (GI: 115487130), Os11g0130300 (GI: 115483977), DmNP_609063 (GI: 19920842), ZmNP_001142328.1 (GI: 226529910), ScNSE1 (GI: 6323035). The National Center for Biotechnology Information accession number for NSE3 showed in this study and the supporting information are At1g34770 (AtNSE3, GI: 79588566), HSMAGE_G1 (GI: 20162572), HSMAGE_A4 (GI: 58530865), Os07g0151300 (GI: 115470609), SpNSE3 (GI: 6320494), ScNSE3p (GI: 6320494), BtMAGE_G1 (GI:118151240). (C) The conserved domain in AtNSE1 and AtNSE3. AtNSE1 contains SMC_Nse1 domain and a RING-like domain. The SMC_Nse1 contain two winged-helix domains. tNSE3 contain a MAGE (melanoma antigen) domain which contains two winged-helix (WH) domains. (D-E) The 3D structure of AtNSE1 (D) and AtNSE3 protein (E) modeled by SWISS-MODEL.

Fig. S3

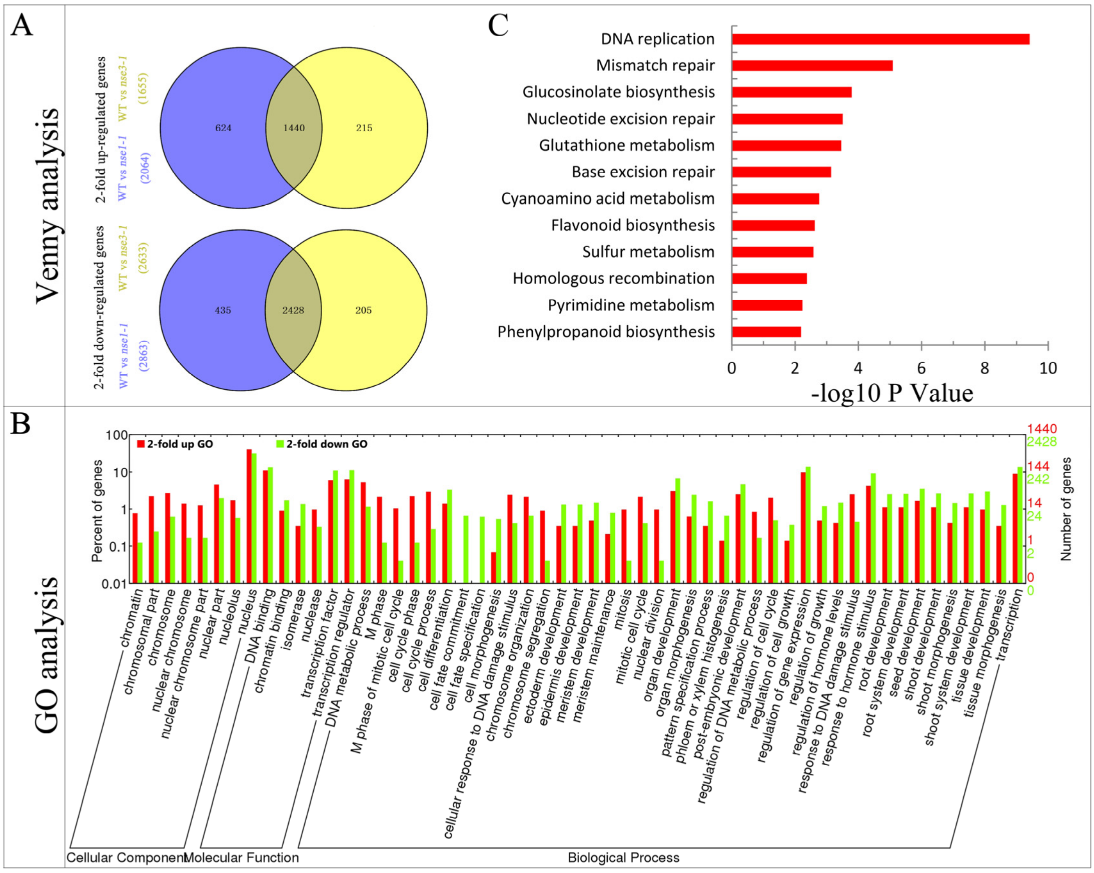

Fig. S3 RNA-seq analysis of wild-type, *nse1-1* and *nse3-1* ovule.

(A) Venn diagram showing the differential expression genes of *nse1-1* and *nse3-1* mutants. Wild-type vs *nse1-1* or *nse3-1* showing the differential expression genes between wild-type and *nse1-1*or *nse3-1*, respectively. (B) Histogram presentation of gene ontology (GO) classification based on RNA-seq data. The red histograms show up-regulated genes in both *nse1-1* and *nse3-1* (showing as 2-fold up GO), and the green histogram show down-regulated genes in both *nse1-1* and *nse3-1* (showing as 2-fold down GO). (C) KEGG pathway enrichment analysis of up-regulated genes in the mutants' ovules. The P value indicates the significance of the correlation between the pathways.

Fig. S4

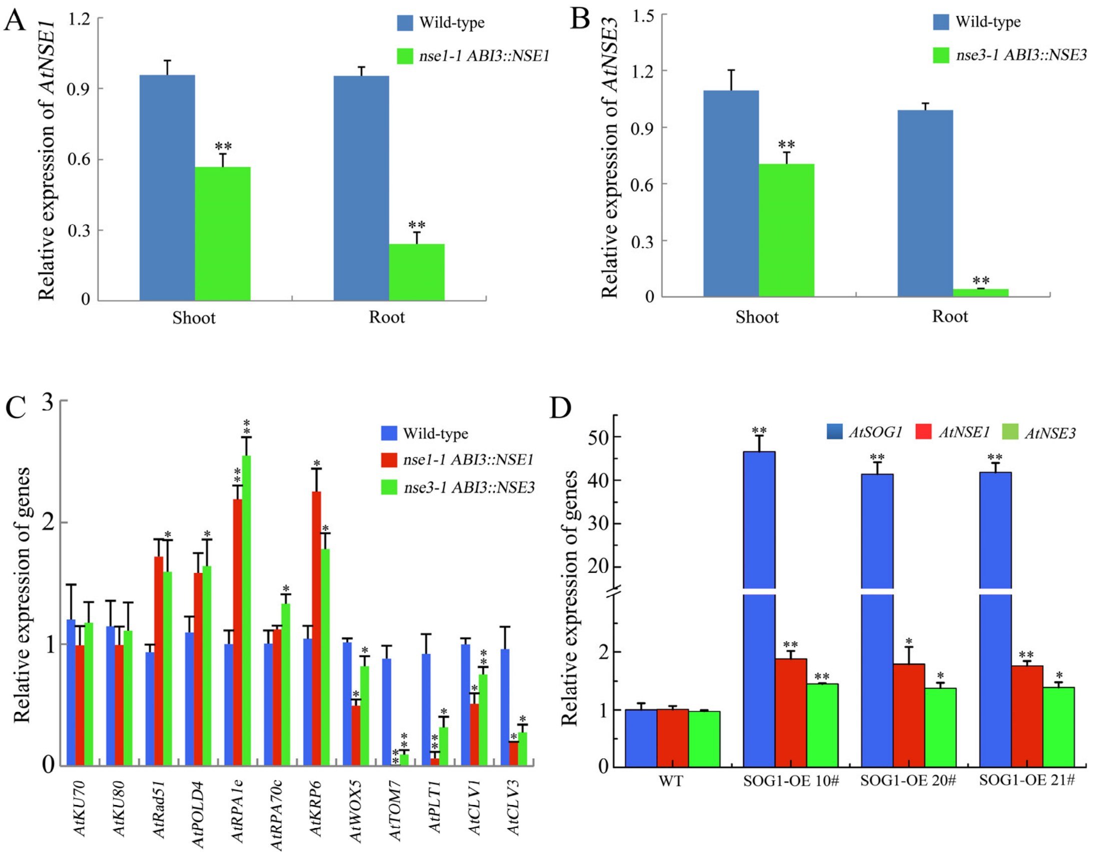

Fig. S4 Relative expression of genes in wild type, the partly complemented mutants and *AtSOG1* over-expressed lines.

(A-B) Relative expression of *AtNSE1* (A) and *AtNSE3* (B) in different tissues of 10 days old partially complemented mutant seedlings. (C)Real-time quantitative PCR validations for selected genes from the DSBs repair pathways, cell cycle and maintenance of RAM and SAM in the 2-week old partially complemented mutant seedlings. (D) Relative expression level of *AtNSE1* and *AtNSE3* in different *AtSOG1* over-expression (OE) transgenic lines. 2-week old seedlings are used to be detected. The asterisks indicate a statistically significant difference according to Student's t-test ($p<0.05$ for single asterisk, and $p<0.01$ for double asterisks).

Fig S5

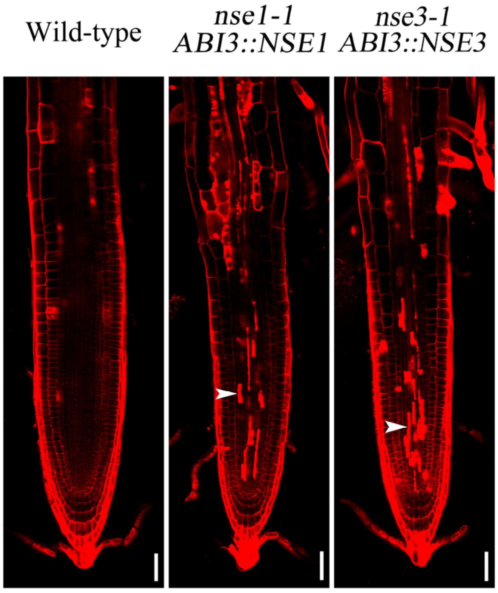

Fig. S5 Propidium iodide staining analysis of root tips in wild-type, *nse1-1 ABI3::NSE1* and *nse3-1 ABI3::NSE3* seedlings.

All seedlings were cultured on 1/2 MS plate for 5 days. Red cells stained with propidium iodide were dead and showed by white arrows. Bars=50 μm.

Supplementary Tables:

Table S1. Primers (5' to 3') used in the experiments.

3.1 Primers for mutant verification

	FP	RP
1-1	gcaagaaaagggaatgacaaagg	gctttttggcaggtcctttttaa
1-2	tacttgtgtatccggacaggg	cccttttcttgctatgtgtgc
3-1	cggtctcttacgacgatcttttt	ttctcgctaacttgaggatctagc
3-2	ttacgatctttttggtttcggt	aaggccaaatcttcctctaatcat
CSLB	cccatttggacgtgaatgtagacac	
pAC161LB	atctgatttcccaacccaatc	
LBb1.3	attttgccgatttcggaac	

3.2 Primers for complementation

	FP	RP
AtNSE1-ORF	accTCTAGAatggcatcgctaagctggaag	accGGATCCcctcgagtgcccaacgctac
AtNSE3-ORF	cgGGATCCatggccgacgaagaagat	catgCCATGGCatcattaagctctacaactg ctac
GFP		agggtcagcttgccgtaggtg

3.3 Primers for subcellular localization fusion constructs

	FP	RP
NSE1-Pro-EGFP	accAAGCTTaattattttttcgctccatattcc	accTCTAGAccttagagacctccgtaatttca c
NSE3-Pro-EGFP	aaCTGCAGgatcaaaaactgctgtcacga	cgGGATCCtctgaaatcaaattcgcgtc

3.4 Primers for in situ hybridization

	FP	RP
AtNSE1-ISH-S	CATAATACGACTCACTATAGGgatttca ctcaatcttcacc	tctctacaagccttcaact
AtNSE1-ISH-AS	atttcactcaatcttcacc	CATAATACGACTCACTATAGGGt ctctacaagccttcaact
AtNSE3-ISH-S	CATAATACGACTCACTATAGGGatggc cgacgaagaagatt	tggagttcctgagctttgg
AtNSE3-ISH-AS	atggccgacgaagaagatt	CATAATACGACTCACTATAGGGt ggagttcctgagctttgg

3.5 Primers for yeast two-hybrid

	FP	RP
AtNSE1-AD	accGGATCCATatggcatcgctaagctggaag	accGAGCTCCcctcgagtgcccaacgctac
AtNSE1-BK	accGGATCCATatggcatcgctaagctggaag	accGTCGACGcctcgagtgcccaacgcta c
AtNSE3-AD	accGGATCCATatggccgacgaagaagat	accGAGCTCCatcattaagctctacaactgc taca
AtNSE3-BK	accGGATCCATatggccgacgaagaagat	accGTCGACGatcattaagctctacaactgc taca

3.6 Primers for partial complement

	FP	RP
NSE1-ABI3	ggGGTACCatggcatcgctaagctggaag	
NSE3-ABI3	ggGGTACCatggccgacgaagaagat	
NOST-CDS		ccgGAATTCccgatctagtaacatagatgacac

3.7 Primers for AtSOG1 over-expression constract

	FP	RP
AtSOG1-ORF	gctctagaatggctgggcgatcatgg	cgagctctcaatcagtctttccagtcccc

3.8 Primers for qRT-PCR

	FP	RP
GAPDH	gagtctactggtgtcttcactg	caaggtcggacttgtattcgtg
AtNSE1-qRT	ttatagaagcaattgcacaggatga	gtcccttacaagttcatcgagagtt
AtNSE3-qRT	gcttcctcagtcacagagttctg	caaagggtttcttcagggatt
KU70	tatggcgatgaccctgatgaa	cgaggagattgttggcagtcag
KU80	aggagccaaagcaattcaatga	ccccagcgttctcgtctactat
POLD4	tgacaaggaggaggagatgct	acacgaccttgccacagaca
RAD51	gagtttggtgtggctgttgttat	aaacatggcgagcttatcactt
RPA1e	aatcgggtcaatctgggaatc	tctcggtagtcctcctccataac
RPA70c	agtcgatgcacgcagagaatt	tttgtgccacccatgtattgt
CLV1	gaggaacacggaagagga	ccacggatttaggagggt
CLV3	aagacagccaagaaacaa	cttaccaaacgaaacaga
PLT1	caccgctggtaataatgt	gtgtccaatgcacgtctt
WOX5	gaatcataaggctaggga	tcaccggaaagagttgta
TMO7	aggcaatcatcaggaact	tgaaaccttgtcggaacg
KRP6	tgggagaaacgacaacagaaat	ttgcttcttcttatcgtcttgact
SOG1-qRT	tctggtttgtcagggtta	aatctttctacgctttcg

Table S2. Transmission of the *AtNSE1* and *AtNSE3* mutants.

Cross (Female×Male)[a]	With T-DNA insertion(W)	Without T-DNA insertion(WO)	W:WO Rate[b]	Expected Rate	TE (Female)[d]	TE (Male)[d]
nse1-1/+ × WT	587	582	1.01:1[c]	1:1	100.9%	NA
WT × *nse1-1/+*	574	609	0.94:1[c]	1:1	NA	94.3%
nse1-2/+ × WT	137	140	0.98:1 [c]	1:1	97.9%	NA
WT × *nse1-2/+*	178	166	1.07:1 [c]	1:1	NA	107.2%
nse3-1/+ × WT	363	338	1.07:1[c]	1:1	107.4%	NA
WT × *nse3-1/+*	396	429	0.92:1[c]	1:1	NA	92.3%
nse3-2/+ × WT	625	604	1.03:1[c]	1:1	103.5%	NA
WT × *nse3-2/+*	392	347	1.13:1[c]	1:1	NA	113.0%

[a]Seeds obtained by each cross were grown on selective plates to determine the segregation for *nse1-1/+, nse3-1/+*, *nse3-2/+*, but for *nse1-2/+*, seeds obtained by each cross were sown on nonselective plates and determined the segregation by PCR. [b]With T-DNA insertion(W):Without T-DNA insertion(WO). [c]Not significantly different from the segregation ratio of 1:1 (P>0.05). [d]TE = Resistant/Sensitive × 100%. NA, not applicable.

Supplementary methods:

Homology Modeling

The sequences of AtNSE1 (TAIR accession number: At5g21140) and AtNSE3 (TAIR accession number: At1g34770) protein were downloaded from the Arabidopsis Information Resource (TAIR, http://www.arabidopsis.org/). Their 3D structures were modeled by using the SWISS-MODEL server (http://swissmodel.expasy.org/, Guex et al., 2009; Biasini et al., 2014). The homology templates were 3nw0.1.A and 3nw0.1.B (Doyle et al., 2010). The PDB files of modeled proteins were download and displayed using Swiss-PdbViewer. The 3D structural images were treated by POV-Ray 3.7 (http://www.povray.org/).

Supplementary Literature Cited:

Biasini M., Bienert S., Waterhouse A., Arnold K., Studer G., Schmidt T., Kiefer F., Gallo Cassarino T., Bertoni M., Bordoli L., Schwede T. (2014) SWISS-MODEL: modelling protein tertiary and quaternary structure using evolutionary information. Nucleic Acids Research 42: W252-W258.

Doyle J.M., Gao J., Wang J., Yang M., Potts P.R. (2010) MAGE-RING protein complexes comprise a family of E3 ubiquitin ligases. Mol.Cell 39: 963-74.

Guex N., Peitsch M.C., Schwede T. (2009) Automated comparative protein structure modeling with SWISS-MODEL and Swiss-PdbViewer: A historical perspective. Electrophoresis 30: S162-S173.
